# A Novel Approach for the Treatment of Recurrent Oroantral Fistula Occurring at an Infected Sinus Augmentation Site

**DOI:** 10.3390/medicina60020343

**Published:** 2024-02-19

**Authors:** Won-Bae Park, Min-Soo Bae, Wonhee Park, Hyun-Chang Lim, Ji-Young Han

**Affiliations:** 1 Department of Periodontology, School of Dentistry, Kyung Hee University, Seoul 02447, Republic of Korea; wbpdds@naver.com; 2 Private Practice in Periodontics and Implant Dentistry, Seoul 02771, Republic of Korea; 3 With Dental Clinic, #401 Dae-oh bldg, 53-1, Yeouinaru-ro, Yeongdeungpo-gu, Seoul 07273, Republic of Korea; bms0421@gmail.com; 4 Department of Prosthodontics, Division of Dentistry, College of Medicine, Hanyang University, 222-1 Wangsimni-ro, Seongdong-gu, Seoul 04763, Republic of Korea; whpark@hanyang.ac.kr; 5 Department of Periodontology, Periodontal-Implant Clinical Research Institute, School of Dentistry, Kyunghee daero 23, Dongdaemoon-gu, Seoul 02447, Republic of Korea; 6 Department of Periodontology, Division of Dentistry, College of Medicine, Hanyang University, 222-1 Wangsimni-ro, Seongdong-gu, Seoul 04763, Republic of Korea

**Keywords:** bone regeneration, complication, dental implant, oroantral fistula, sinus graft infection

## Abstract

Closing a recurrent oroantral fistula (OAF) that occurs at an infected sinus augmentation site is a challenge for clinicians. The recurrent OAF has a detrimental impact on bone regeneration and subsequent implant placement. This case report includes three cases in which sinus graft infection and OAF occurred after maxillary sinus augmentation (MSA). In these cases, treatments to control sinus infection were performed using an otolaryngologist; then, intraoral interventions comprising mucosal flap procedures, bone grafts, and barrier membrane applications were performed 2–5 times by oral surgeons. Nevertheless, OAF recurred persistently. The failure to stop OAF recurrence may be due to the inability to effectively block air pressure at the OAF site. Following a comprehensive debridement of the infected tissue at the previous sinus augmentation site, a pouch was created through sinus mucosal elevation. The perforated sinus mucosa at the OAF site was covered with a non-resorbable membrane in one case and with resorbable collagen membranes in the other two cases, followed by bone grafting within the pouch. Lastly, this procedure was completed by blocking the entrance of the pouch with a cortical bone shell graft and a resorbable collagen membrane. The cortical bone shell graft, obstructing the air pressure from the nasal cavity, facilitated bone formation, and, ultimately, allowed for implant placement. Within the limitations of the present case report, the application of a guided bone regeneration technique involving a cortical bone shell graft and a barrier membrane enabled the closure of the recurrent OAF and subsequent implant placement.

## 1. Introduction

Lateral maxillary sinus augmentation (MSA) enables successful implant placement in the maxillary posterior region with insufficient residual bone height and pneumatized maxillary sinus [1,2]. Various early or late complications after MSA procedures have been reported [3]. Early complications are mostly related to sinus membrane perforation, the spill of bone graft materials, the plugging of the ostium, and sinus graft infection/postoperative sinusitis [4,5]. Late complications occur in cases with delayed sinus graft infection after implant loading when the grafted sinus is infected with peri-implantitis [5].

Postoperative infection after MSA can involve part of the sinus graft or the entire sinus graft, eventually resulting in graft sequestration, implant displacement, paranasal sinusitis, or oroantral fistula (OAF) [3]. Compared to OAF, which occurs at the tooth extraction socket, the OAF at the infected sinus augmentation site is challenging for clinicians because of its recurrent nature. Successful results can be achieved only when the appropriate treatment method is determined and the treatment sequence is well established. However, establishing a proper treatment protocol to handle complex situations is challenging due to the highly diverse nature of cases. In addition, consultation with an otolaryngologist and functional endoscopic sinus surgery (FESS) may be necessary [6].

If implant placement is planned at the recurrent OAF site accompanied by sinus graft infection, reconstruction with bone tissue rather than soft tissue is required. A recent case report showed that the successful closure of the OAF at the tooth/implant extraction site was achieved with an MSA procedure using particulate bone and a resorbable barrier membrane [7]. However, based on empirical observations, the outcome of MSA using particulate bone graft and barrier membrane in cases of recurrent OAF after MSA appears to be unpredictable.

To the best of our knowledge, there have been few reports on the guided bone regeneration (GBR) technique using a cortical bone shell graft and barrier membrane in cases of recurrent OAF with sinus graft infection. The purpose of this case report is to introduce a novel technique for closing a recurrent OAF that occurred at the site of sinus graft infection after MSA and GBR, in preparation for future implant placement.

## 2. Case Presentation

This case report includes three patients who were referred to our clinic after multiple attempts to close an OAF at the infected sinus augmentation site using particulate bone grafts, barrier membranes, and mucosal flaps.

### 2.1. Case 1

**Figure 1:**
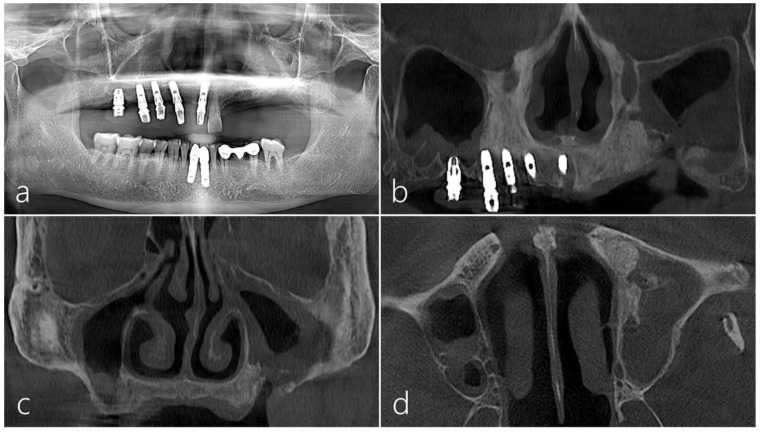
Case 1: (**a**) The preoperative panoramic radiograph showed a bone deficiency on the left maxillary posterior region. (**b**) The preoperative coronal cone-beam computed tomography (CBCT) image revealed a substantial loss in grafted bone after previous maxillary sinus augmentation. (**c**) On the coronal CBCT image, the mucosa of the left maxillary sinus was significantly thickened, and the natural ostium was observed to be closed. (**d**) The preoperative axial CBCT image showed remnants of augmented bone grafts in the left maxillary sinus.

This patient was a 57-year-old male smoker. He did not have any systemic diseases that could interfere with the operation. The patient had an OAF in the maxillary left posterior region. After reviewing the patient’s history, a panoramic radiograph and a cone-beam computed tomography (CBCT) were conducted (Figure 1a,b). Two years previously, in the referred clinic, he underwent a dental implant placement procedure in the right maxillary posterior region without MSA. However, a final restoration was not delivered due to continuous bone resorption. MSA was performed on the pneumatized maxillary sinus in the left upper posterior region. However, a sinus graft infection occurred. After incision and drainage (I&D), the infection decreased, but an OAF developed. Despite five attempts made to close the OAF in several clinics, the fistula remained unclosed. When the patient was referred to our clinic, we consulted with an otolaryngologist. The patient was prescribed antibiotics (amoxicillin/clavulanate potassium 375 mg, three times a day) for 2 weeks.

Preoperative panoramic radiography showed a deficiency of the maxillary left edentulous ridge (Figure 1a). A significant portion of the sinus graft that had been previously augmented was observed to be lost in the preoperative CBCT scan (Figure 1b). The mucosa of the left maxillary sinus was significantly thickened and the natural ostium was found to be closed (Figure 1c). Some remnants of the previous sinus bone graft were observed in the left maxillary sinus (Figure 1d).

**Figure 2:**
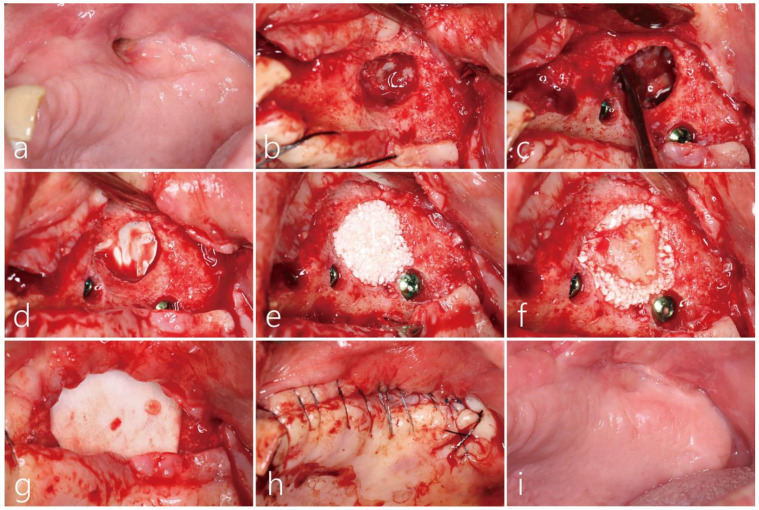
(**a**) The preoperative clinical view exhibited a recurrent oroantral fistula (OAF) on the buccal vestibular side of the left maxillary posterior area. (**b**) After reflecting the mucoperiosteal flap, a bone defect with a diameter of about 1.7 cm was observed. (**c**) A pouch was formed by elevating the scar-like tissue in the left sinus. (**d**) The perforated sinus mucosa at the OAF site was protected with a properly trimmed titanium-reinforced, high-density polytetrafluorethylene (d-PTFE) membrane. (**e**) The elevated space was filled with synthetic bone graft substitutes. (**f**) The cortical bone shell graft, obtained from the right maxillary sinus window, was positioned without any fixation. (**g**) The grafted bone was covered with a resorbable collagen membrane. (**h**) The surgical site was closed using 5-0 nylon. (**i**) The recurrent OAF was successfully closed.

After removing the implants placed on the right side, an MSA was performed using a lateral window approach and the implants were replaced simultaneously in the right maxillary posterior area. A recurrent oroantral fistula was observed on the buccal vestibular area of the left maxillary posterior area (Figure 2a), reaching the left maxillary sinus without resistance during insertion with the implant depth instrument. Air leakage was observed during the nose blowing test. After reflecting the mucoperiosteal flap, a bone defect with a diameter of about 1.7 cm was observed. The exposed mucosa of the left maxillary sinus appeared thick, resembling firm scar tissue (Figure 2b). The perforated sinus mucosa was carefully separated and elevated from the sinus floor using a sinus elevation kit (Genoss Co., Ltd., Suwon, Republic of Korea). A pouch was formed within the maxillary sinus, creating a space (Figure 2c). Due to the predominance of scar tissue in the elevated sinus mucosa, creating space became exceedingly challenging. Therefore, the perforated sinus mucosa was protected with an appropriately trimmed and contoured titanium-reinforced high-density polytetrafluoroethylene (d-PTFE) membrane (Cytoplast™, Osteogenics Biomedical, Inc., Lubbock, TX, USA) (Figure 2d). After preparing implant sites, four SLA-textured implants (Implantium^®^, Dentium Co., Ltd., Suwon, Republic of Korea) were utilized (Figure 2d). The elevated space was filled with a synthetic bone graft substitute (Osteon II™, Genoss Co., Ltd., Suwon, Republic of Korea) (Figure 2e). The lateral sinus window bone, obtained during simultaneous lateral MSA on the contralateral side, was used as the cortical bone shell graft. The cortical bone shell graft was passively positioned (Figure 2f) and then covered with a resorbable collagen membrane (Figure 2g). Finally, the surgical site was closed using 5-0 nylon (Figure 2h). Postoperatively, the patient was prescribed systemic antibiotics (amoxicillin/clavulanate potassium 375 mg, three times a day) for 2 weeks, and strict avoidance of nose blowing and smoking was emphasized. The sutures were removed after 2 weeks, and the healing was uneventful until the uncovering procedure six months later. The recurrent OAF was successfully closed (Figure 2i).

**Figure 3:**
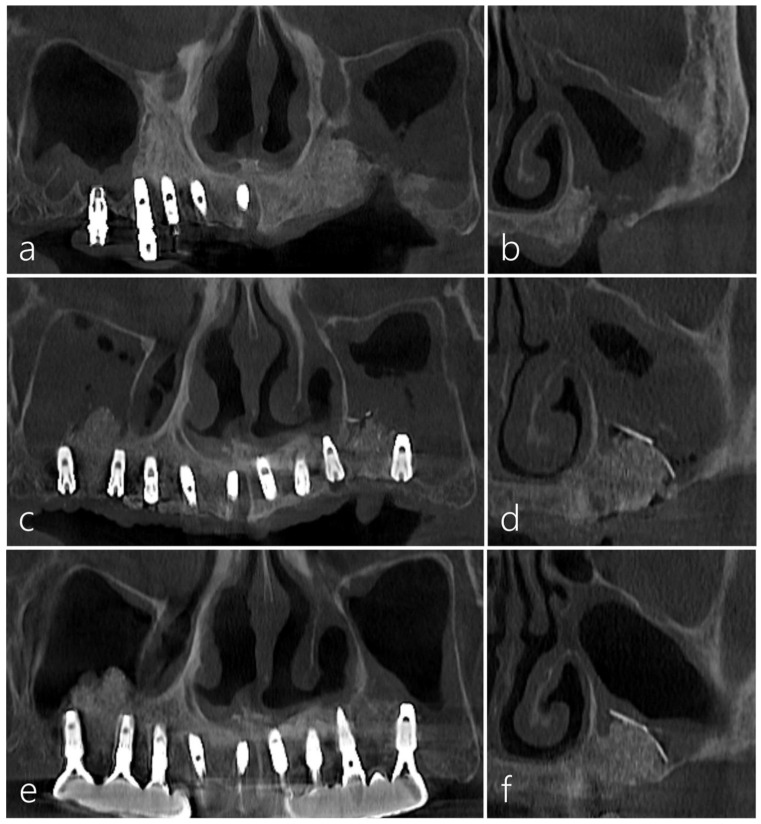
(**a**) The preoperative CBCT image showed a significant loss in the augmented bone graft in the left maxillary posterior area. (**b**) Sinus mucosal thickening was observed in the left maxillary sinus. (**c**,**d**) The augmented bone graft and titanium-reinforced d-PTFE membrane were observed in the panoramic and coronal CBCT scans taken immediately after the procedure. (**e**) In the CBCT image taken 12 months after the final prosthesis was delivered, reduced sinus mucosal thickening was observed. (**f**) The coronal CBCT image showed the well-maintained titanium-reinforced d-PTFE membrane on the right maxillary sinus.

The preoperative panoramic and coronal images of CBCT revealed a significant loss in the augmented sinus graft and sinus mucosal thickening on the left maxillary sinus (Figure 3a,b). In the panoramic and coronal CBCT scans taken immediately after the procedure, a well-augmented sinus graft and titanium-reinforced d-PTFE membrane were observed (Figure 3c,d). The CBCT scans 12 months after the prosthesis delivery revealed the reduced mucosal thickening and well-maintained sinus graft and d-PTFE membrane without complications (Figure 3e,f). There was no exposure of the d-PTFE membrane into the maxillary sinus, indicating successful coverage and integration.

### 2.2. Case 2

This patient was a 43-year-old male non-smoker. He was taking antihypertensive drugs and did not have any other underlying medical conditions. The patient was referred to our clinic due to a severe sinus graft infection and recurrent OAF in February 2012. Panoramic radiography and CBCT scans taken during the patient’s previous treatment were provided by the referring clinic.

**Figure 4:**
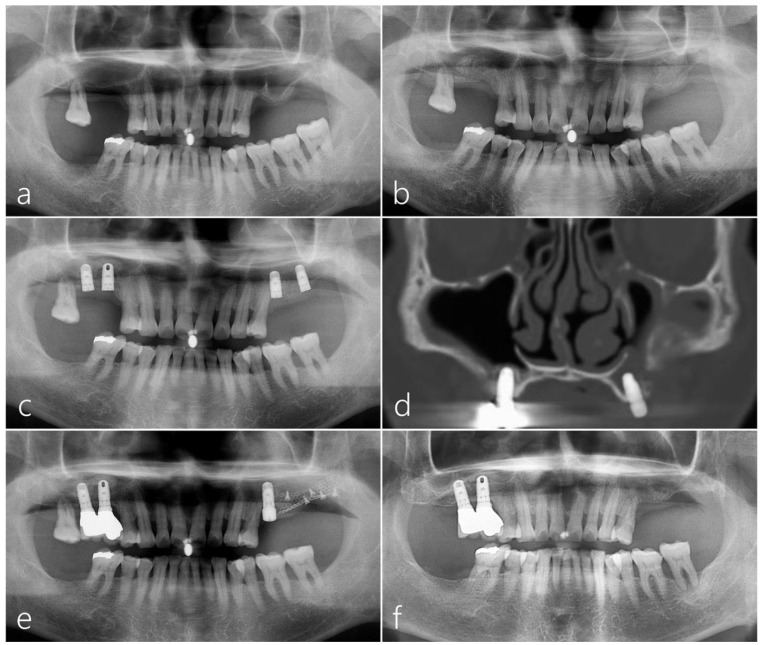
Case 2: (**a**) The panoramic radiography from the referred clinic showed pneumatized maxillary posterior area on both sides. (**b**) The maxillary sinus augmentation was performed at the referred clinic for implant placement. (**c**) Six months later, two HA-coated implants were placed on each side at the referred clinic. (**d**) The coronal CBCT scan showed a buccal bone defect with opacification of the left maxillary sinus. (**e**) The panoramic radiograph taken immediately after the revision procedure showed the augmented bone graft with titanium mesh and pins. (**f**) The panoramic radiograph taken when the patient was referred to our clinic revealed that the augmented bone graft had disappeared at the oroantral fistula site.

At the referred clinic, the patient underwent MSA for implant placement in the maxillary left and right posterior areas in 2007 (Figure 4a,b). Subsequently, two HA-coated implants (4.8 × 10 mm Zimmer TSV™, Zimmer Biomet Dental, Warsaw, IN, USA) were placed on each side after 6 months (Figure 4c). However, following implant placement, a sinus graft infection occurred and progressed to maxillary sinusitis on the left side. Subsequently, upon the removal of the implant placed at the #27 site, an OAF was developed. Despite continuous use of antibiotics and several surgical interventions, the size of the defect in the ridge crest gradually increased. The coronal CBCT scan revealed a buccal bone defect and sinus opacification on the left maxillary sinus (Figure 4d). Ultimately, the patient was referred to an otolaryngologist at the university hospital and underwent FESS treatment. After FESS, the patient’s clinical symptoms improved. In October 2008, a revision surgery using titanium mesh was attempted for the reconstruction of the missing ridge crest and closure of the OAF; however, unfortunately, it was not successful (Figure 4e). Another implant placed in the #25 site was explanted. Subsequently, the patient was referred to our clinic in February 2012 (Figure 4f). We had the patient consult with an otolaryngologist before surgery. The patient was prescribed a two-week course of antibiotics (amoxicillin/clavulanate potassium 375 mg, three times a day).

**Figure 5:**
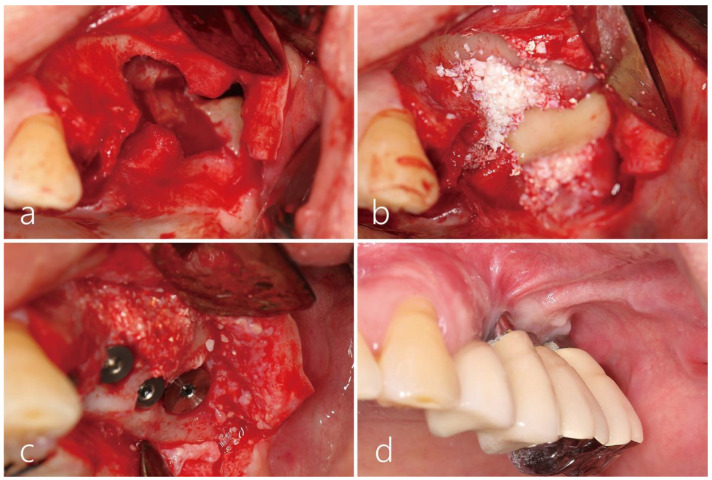
(**a**) After reflection of the mucoperiosteal flap, a significant amount of buccal bone loss was observed, including the ridge crest. (**b**) The perforation of the maxillary sinus mucosa was repaired with a resorbable collagen membrane. The created space (pouch) was filled using a particulate bone graft and a cortical bone shell graft obtained from the ascending ramus. (**c**) Reentry was performed for implant placement after 6 months. Extensive bony defects in the buccal area were successfully reconstructed. (**d**) After 6 months, the uncovering surgery was performed, followed by the delivery of the prosthesis.

In May 2012, a guided bone regeneration technique using particulate bone, a cortical bone shell graft, and a barrier membrane was performed to reconstruct the missing ridge crest and close the OAF. Under local anesthesia, mucoperiosteal flaps were reflected on the buccal and palatal sides. A significant amount of the buccal bone, including the ridge crest, was observed to be lost (Figure 5a). Due to the risk associated with achieving implant stability, delayed implant placement was planned. The maxillary sinus mucosa had healed with scar-like tissue, appearing firm and thick. Consequently, an appropriate amount of this tissue was removed using a #15 Bard Parker blade. Adequate osseous housing was necessary to ensure effective blood supply from the sinus wall and stabilize bone graft substitutes within the wound. Hence, the sinus mucosa was meticulously detached from the sinus floor using various types of curettes, and a pouch within the maxillary sinus was created through additional elevation. Perforation of the sinus mucosa was repaired using a resorbable collagen membrane (Bio-Gide^®^ Geistlich Pharma AG, Wolhusen, The Switzerland), and the pouch was subsequently filled with particulate bone graft (Bio-Oss^®^, Geistlich Pharma AG, Wolhusen, The Switzerland). Because the buccal defect was very large, the cortical bone shell graft obtained from the ascending ramus on the ipsilateral side was placed passively without the use of screw fixation (Figure 5b). Finally, after covering it with a resorbable collagen membrane (Bio-Gide^®^ Geistlich Pharma AG, Wolhusen, Switzerland), the flap was secured closed. The patient was prescribed antibiotics (ciprofloxacin 500 mg, Ildong Pharmaceutical Co., Seoul, Republic of Korea) and a nonsteroidal anti-inflammatory drug for 2 weeks. During this period, the patient was instructed to refrain from blowing his nose. The surgical site healed uneventfully without any complications. After 6 months, a reentry procedure was performed for implant placement. The buccal mucoperiosteal flap was reflected and the extensive buccal bone defect was successfully filled with bone-like tissue. Four HA-coated implants (Zimmer TSV™, Zimmer Biomet Dental, Warsaw, IN, USA) were placed (Figure 5c). Six months after implant placement, the uncovering procedure was performed, followed by the delivery of the prosthesis. Scar tissue was observed on the buccal side due to multiple surgeries; however, revision surgery was not performed because the patient reported no discomfort (Figure 5d).

**Figure 6:**
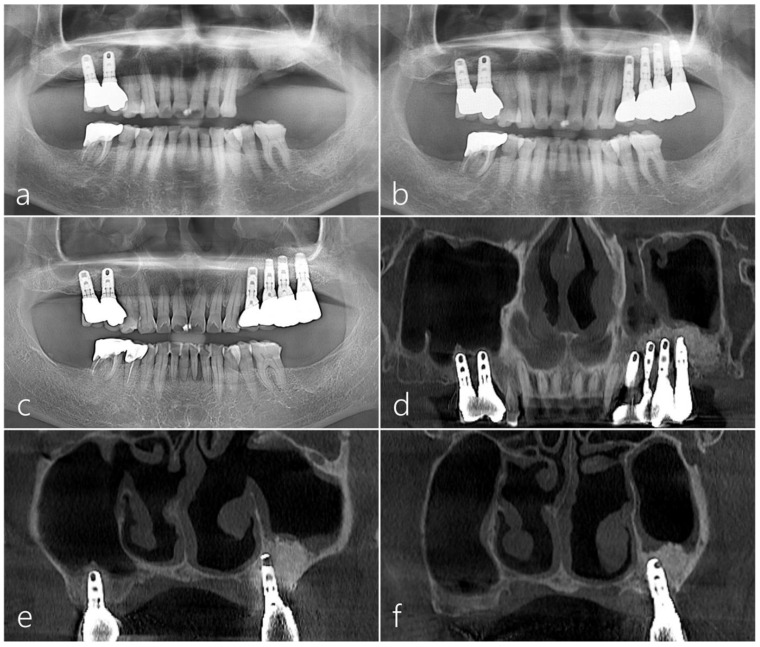
(**a**) Panoramic radiography taken immediately after maxillary sinus augmentation (MSA) using a particulate bone graft and a cortical bone shell showed radiopaque bone density in the left maxillary sinus. (**b**) Four implants were placed 6 months after MSA, followed by the delivery of the prosthesis 6 months after the implant placement. (**c**) Panoramic radiography 5 years after the delivery of the prosthesis. (**d**–**f**) On the panoramic and coronal images of the CBCT taken 5 years after the prosthesis was delivered, the left sinus showed no significant mucosal thickening and maintained bone integrity.

Panoramic radiography conducted immediately after MSA using a cortical bone shell and particulate bone graft showed radiopaque bone density in the left maxillary sinus (Figure 6a). Four implants were placed 6 months after MSA, and the prosthesis delivery was performed 6 months after the implant’s placement (Figure 6b). Panoramic radiography taken five years after the prosthesis delivery showed well-maintained augmented bone and implants in the left posterior area (Figure 6c). On the panoramic and coronal images of the CBCT taken 5 years after prosthesis delivery, the left sinus showed no significant mucosal thickening, and the augmented bone was well-maintained (Figure 6d–f).

### 2.3. Case 3

**Figure 7:**
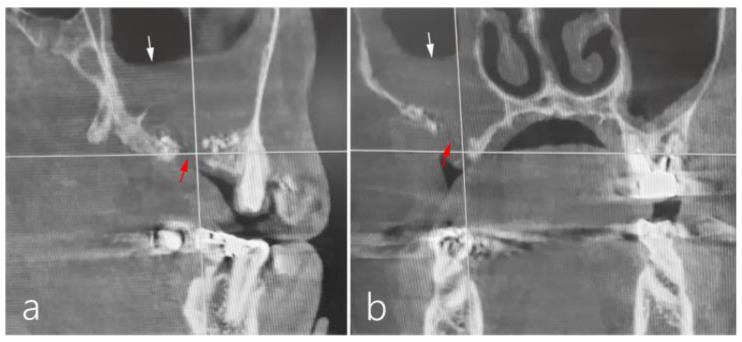
Case 3: (**a**) In the preoperative sagittal image of CBCT, the previous augmented bone graft had mostly disappeared, and there was severe sinus mucosal thickening (white arrow). (**b**) A bone defect extending from the ridge crest to the buccal side (red arrow) was observed on the preoperative coronal CBCT.

The patient was a 66-year-old female non-smoker with diabetes mellitus. In the referred clinic, she underwent MSA using a lateral approach. However, three weeks after MSA, severe edema and pus discharge were observed, leading to an I&D procedure in the vestibular area. Additionally, antibiotics were prescribed for three weeks by an otolaryngologist. The patient’s symptoms improved; however, an OAF developed. Despite three attempts to close OAF using bone grafts and a barrier membrane, the OAF persisted. On the sagittal image of CBCT, the previous augmented bone graft had mostly disappeared, and there was severe sinus mucosal thickening (Figure 7a). A bone defect extending from the ridge crest to the buccal side was observed on the coronal CBCT image (Figure 7b).

**Figure 8:**
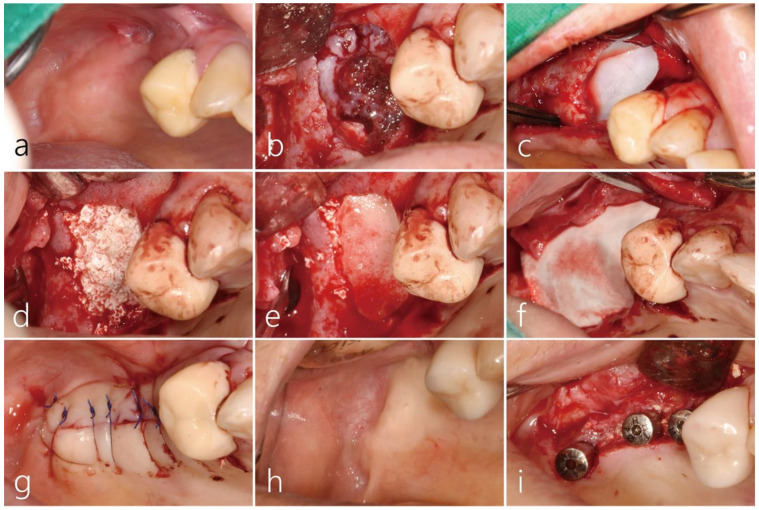
(**a**) The preoperative clinical photo showed an oroantral fistula (OAF) on the buccal side of the right posterior area. (**b**) After the reflection of the buccal flap, the infected bone graft with the surrounding granulation tissue was shown. (**c**) The perforated sinus mucosa was covered with a resorbable collagen membrane. (**d**) The pouch was filled with particulate bone graft substitutes. (**e**) The entrance to the pouch was blocked with a cortical bone shell graft obtained from the lateral sinus window on the contralateral side. (**f**) Finally, the grafted bone was covered with a resorbable collagen membrane. (**g**) The mucoperiosteal flap was closed with 5-0 nylon. (**h**) Six months after surgery, healing was uneventful, and an OAF was closed. (**i**) The OAF site was filled with bone-like tissue and three implants were placed.

Due to severe sinus mucosal thickening, we had the patient consult with an otolaryngologist. Nasal irrigation with a two-week course of antibiotics (amoxicillin/clavulanate potassium 375 mg, three times a day) was performed. Her glucose and HbA1c levels were well controlled. Before surgery, an OAF was observed on the buccal side of the right posterior area (Figure 8a). Under local anesthesia, the buccal mucoperiosteal flap was reflected. A very wide bony defect (1.5 × 2.0 cm) was observed, and the sinus membrane exhibited a proliferation of inflammatory tissue around the OAF. Debridement was performed on the infected bone graft and the surrounding granulation tissue (Figure 8b). The sinus mucosa was carefully separated from the sinus floor and then further elevated, forming a pouch within the maxillary sinus. The inflammatory exudate within the maxillary sinus was meticulously suctioned through the OAF site, and thorough saline irrigation was performed. The OAF site at the top of the pouch was covered with a resorbable collagen membrane (Genoss Co., Ltd., Suwon, Republic of Korea) (Figure 8c), and the pouch was filled with a particulate bone graft substitute (Osteon II™, Genoss Co., Ltd., Suwon, Korea) (Figure 8d). The entrance of the pouch was covered with a cortical bone shell graft harvested from the contralateral sinus window (Figure 8e). Finally, the surgical site was covered with a resorbable collagen membrane (Genoss Co., Ltd., Suwon, Republic of Korea) (Figure 8f). The mucoperiosteal flap was closed with 5-0 nylon (Figure 8g). The patient was prescribed antibiotics (ciprofloxacin 500 mg, Ildong Phamaceutical Co, Seoul, Korea) and an analgesic anti-inflammatory drug for 2 weeks. Additionally, the patient was advised not to blow her nose. The surgical site healed uneventfully and the OAF was successfully closed (Figure 8h). Six months later, the mucoperiosteal flap was reflected for implant placement, and the previous wide defect was filled with bone-like tissue. Three implants (Ø 4.0 × 10 mm, Osstem implant, Seoul, Republic of Korea) were placed. Because the initial stability was good, the healing abutment was connected to the implant (Figure 8i). The prosthesis was delivered five months later.

**Figure 9:**
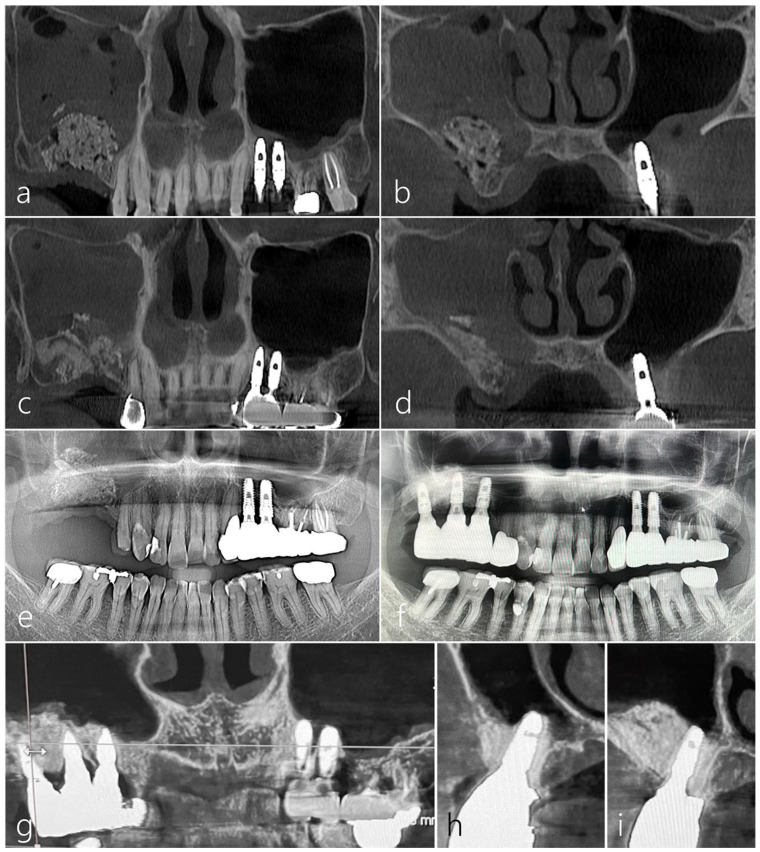
(**a**) The panoramic image of the CBCT taken immediately after surgery revealed an augmented bone graft in the right sinus. (**b**) On the coronal image of the CBCT, an augmented bone graft was observed in the buccal bone defect around the OAF site. (**c**) The panoramic image of CBCT taken 6 months later showed well-maintained augmented bone height. (**d**) On the coronal image of the CBCT taken 6 months later, some contraction of the bone graft area was observed, and the maxillary sinus was completely opacified. (**e**) Panoramic radiography conducted 6 months after bone grafting showed well-maintained augmented bone. (**f**) In panoramic radiography conducted 12 months after the prosthesis was delivered, sufficient bone graft was observed around the implants. (**g**) The CBCT images taken 12 months after the prosthesis delivery showed significantly reduced mucosal thickening on the right maxillary sinus. (**h**,**i**) On the CBCT images taken 12 months after the prosthesis was delivered, a well-maintained augmented bone around the implants was shown.

The panoramic image of the CBCT taken immediately after surgery revealed an augmented bone graft in the right sinus (Figure 9a). On the coronal image of the CBCT, an augmented bone graft was observed in the buccal bone defect around the OAF site (Figure 9b). The surgical site healed uneventfully 6 months after surgery. The panoramic image of CBCT taken 6 months later showed a well-maintained augmented bone height (Figure 9c). However, on the coronal image of the CBCT taken 6 months later, some contraction of the bone graft area was observed, and the maxillary sinus was completely opacified (Figure 9d). The patient had no symptoms due to sinus opacification; moreover, fortunately, sinus opacification did not affect the consolidation of the bone graft within the pouch (Figure 9e). Therefore, additional sinus drainage was performed by forming a small opening hole at the top of the pouch. During implant site preparation, the bone density and quality of the augmentation site were satisfactory. Three Ø 4.0 × 10 mm implants were placed at the #15, #16, and #17 sites. Postoperative healing was uneventful. Uncovering was performed 6 months later. The prosthesis was delivered 2 months later (Figure 9f). The CBCT images taken one year after the prosthesis delivery showed significantly reduced mucosal thickening on the right sinus (Figure 9g) and well-maintained augmented bone around the implants (Figure 9h,i).

## 3. Discussion

An OAF that occurs at an infected sinus augmentation site differs from an OAF that occurs at a tooth/implant extraction socket in terms of the treatment method, treatment difficulty, treatment period, and recurrence. For the successful closure of an OAF occurring at the infected sinus augmentation site and subsequent implant placement, bone regeneration of the newly formed pouch must be achieved through sinus mucosal elevation along with the removal of inflammatory granulation tissue and bone graft particles. The GBR technique using particulate bone graft/cortical bone shell graft and a barrier membrane effectively facilitated bone reconstruction for the defect of an OAF site.

An OAF refers to a situation in which the oral cavity and maxillary sinus communicate with each other as a result of the failure of primary healing of an OAF, dental infections, osteomyelitis, radiation therapy, trauma, or iatrogenic complications [8]. Closing an OAF is crucial to prevent food and saliva contamination in the sinus, which could otherwise result in bacterial infection, impaired healing, and chronic sinusitis [9]. There has been limited research on OAF caused by sinus graft infection. According to several case reports, an OAF caused by sinus graft infection is reported to be accompanied by complications such as graft sequestration, sinus mucosal thickening, sinus opacification, and paranasal sinusitis [5,6]. This study included three cases of sinus graft infection. One case presented with maxillary sinusitis, while the other two cases exhibited severe sinus mucosal thickening.

The successful treatment of an OAF occurring at an infected sinus augmentation site necessitates multiple procedures, including transnasal treatment [6]. Consultation or treatment by an otolaryngologist should precede the treatment of OAFs [6,8,10]. Additionally, control of sinus inflammation must be achieved before OAF closure. This is because the resorbable barrier membrane in contact with the inflamed sinus mucosa can rapidly decompose, potentially disturbing ostial patency. In the present cases, one case (case 2) was preceded by FESS, and two cases were prescribed antibiotics for two weeks before surgery by the otolaryngologist. After the resolution of the sinus infection, appropriate intraoral intervention was performed.

The approach to closing an OAF is primarily determined by the size of the fistula. An OAF occurring in the tooth extraction socket naturally closes without any treatment when the defect is less than 3 mm in width and without epithelialization [9]. An OAF wider than 3 mm or with epithelialization can be closed using various techniques, including an advanced flap, a buccal fat pad, and a palatal rotation pedicle flap [9,11,12,13]. However, an OAF resulting from sinus graft infection often recurs when closure is attempted solely through the conventional advanced flap. Additionally, placing an implant at the OAF site is challenging for clinicians.

Therefore, the need arose for a novel and better technique to treat an OAF resulting from a sinus graft infection. Several authors have reported on a pouch technique with sinus floor elevation [7,8,14]. Additionally, the GBR procedure was applied to the pouch formed within the maxillary sinus [7,8,14]. The perforated sinus mucosa within the pouch was covered with a resorbable collagen membrane, and the pouch was filled with a gelatin sponge, platelet-rich fibrin, or particulate bone graft substitute [8,14,15]. The pouch entrance was covered with a resorbable collagen membrane [7]. Ultimately, the pouch technique is appropriate in situations where the bone graft is surrounded on both sides by a barrier membrane. It has been reported that good clinical and radiological results were achieved with this technique [7,15]. The pouch technique is similar to the sandwich technique [8,15]. However, in our experience, numerous failures were observed when applying the sandwich protocol to recurrent OAFs occurring at infected sinus augmentation sites. The three present cases had also undergone treatment with the sandwich technique multiple times before being referred to our clinic. The causes of failure are presumed to be attributable to the defect’s excessive size and the limited residual bone, which was insufficient to block the air pressure transmitted from the nasal cavity. Recently, ultrasound imaging modalities have been reported to enable the evaluation of the healing process after surgery [16]. It is suggested that ultrasonography allows for more accurate and reliable monitoring of the epithelialization process compared to visual assessment [17].

In the three present cases, OAF closure involves four steps: resorbable collagen membrane/PTFE membrane, particulate bone graft, cortical bone shell graft, and resorbable collagen membrane. Compared to the previously reported case [7], a cortical bone shell graft was added. In case 1, a titanium-reinforced d-PTFE membrane was used instead of a resorbable collagen membrane to provide support to the sinus mucosa and prevent the influx of air pressure. The titanium-reinforced d-PTFE membrane is a non-resorbable barrier membrane typically used with the intention of removal. However, in case 1, it was used with the specific intent of not being removed. This was due to the sinus mucosa being composed of dense and thick scar tissue, a consequence of prolonged infection and repeated surgical procedures. During the 12-month follow-up, the patient reported no symptoms and there was no recurrence of an OAF. Additionally, sinus mucosal thickening diminished, and the CBCT images indicated ostial patency. Nonetheless, covering the perforated sinus membrane with a non-resorbable d-PTFE membrane at the oroantral fistula site is deemed a highly risky procedure with insufficient supporting evidence. To the best of our knowledge, there have been no reported cases utilizing a d-PTFE for covering an OAF as of now. If the sinus mucosa is thin or soft, there is a risk of sinusitis due to exposure, and, therefore, the use of a d-PTFE membrane should be avoided. If infection occurs due to exposure, it is necessary to undergo FESS by an otolaryngologist.

There are reports indicating the successful closure of OAF using bone grafts [4,18,19,20]. In the present cases, cortical bone shell grafts were harvested from the lateral sinus window on the contralateral side or from the ascending ramus on the ipsilateral side. The lateral sinus window offers the advantage of being obtainable in a form similar to that of the OAF site. There is a report that the lateral sinus window is used as a donor for intraoral autografts [21]. An autogenous block bone has the ability to promote new bone formation by releasing osteopromotive molecules [22,23], a process akin to bone formation observed during lateral sinus window repositioning in MSA. The lateral window repositioning procedure has been clinically, histologically, and radiologically validated [22,24,25,26]. Moreover, autogenous block bone has the capability to decrease the air pressure transmitted from the nasal cavity to the sinus cavity. It is suggested that air pressure can exert force on the sinus bone graft, potentially causing the recurrence of OAF. Therefore, we recommended that patients refrain from blowing their nose for two weeks after surgery. While there is a controversy about the influence of smoking on treatment outcomes [27,28,29], the patient in case 1 was advised to refrain from smoking after surgery.

This study is limited by the small number of cases, leading to a lack of robust evidence. Nevertheless, if further cases are undertaken to close an OAF recurring at sinus augmentation sites according to the procedure described here, those further cases may contribute to the revision of clinical guidelines in the future.

## 4. Conclusions

Within the limitations of this case report, in a recurrent OAF occurring at an infected sinus augmentation site, the application of a GBR technique incorporating a cortical bone shell graft and a barrier membrane facilitated the closure of the OAF, allowing for subsequent implant placement.

## Data Availability

Data are contained within the article.

## Abbreviation

OAF: oroantral fistula; MSA, maxillary sinus augmentation; FESS, functional endoscopic sinus surgery; CBCT, cone-beam computed tomography; I & D, incision and drainage; d-PTFE, high-density polytetrafluoroethylene; GBR, guided bone regeneration.
